# Implementing Learning from Excellence in a postanaesthesia care unit: a qualitative study of healthcare professionals’ experiences after six months

**DOI:** 10.1186/s12913-025-12626-8

**Published:** 2025-04-02

**Authors:** Gørill Birkeli, Ellen Catharina Tveter Deilkås, Randi Ballangrud, Anne Karin Lindahl

**Affiliations:** 1 Division of Surgery, Akershus University Hospital, Sykehusveien 25, 1478 Lørenskog, Norway; 2 Department of Health Management and Health Economics, University of Oslo Faculty of Medicine, Institute of Health and Society, Forskningsveien 3A, 0373 Oslo, Norway; 3 Health Services Research Unit, Akershus University Hospital, Sykehusveien 25, 1478 Lørenskog, Norway; 4 Department of Quality Improvement and Patient Safety, Norwegian Directorate of Health, Vitaminveien 4, 0483 Oslo, Norway; 5 Faculty of Medicine and Health Sciences, Department of Health Sciences, Norwegian University of Science and Technology, Teknologivegen 22, Gjøvik, Norway

**Keywords:** Adverse events, Focus groups, Learning health system, Patient safety climate, Patient safety culture, Postoperative care, Quality improvement, Resilience, Safety management

## Abstract

**Background:**

Prevailing efforts to ensure patient safety have primarily focused on learning from errors and adverse events (Safety-I). However, it is advocated that complex systems also learn from success (Safety-II) and focus on healthcare professionals’ well-being at work to increase resilience. Learning from Excellence is a British initiative designed to learn from successful practices and provide positive feedback to the staff. It has gained enthusiastic followers in several countries, including Norway. However, how it influences learning, well-being and quality improvement, has not been studied in-depth. This study intends to address these gaps. Thus, this study aimed to describe healthcare professionals’ experiences with Learning from Excellence six months after its implementation in a postanaesthesia care unit.

**Methods:**

A qualitative descriptive design was applied. Learning from Excellence was implemented in a postanaesthesia care unit of a Norwegian university hospital between November 2022 and May 2023. Six semi-structured focus group interviews were conducted, from May to June 2023, with a convenience sample of nurses (*n* = 17) and physicians (*n* = 7). The data were analysed through inductive reflexive thematic analysis. The study adhered to the COREQ guidelines.

**Results:**

This study reports four prominent themes encapsulating healthcare professionals’ experiences with Learning from Excellence. These themes were termed as follows: 1) ‘Facilitates a more positive working climate’, including sub-themes ‘Helps spread positive feedback’ and ‘Feeling seen and motivated’; 2) ‘Why don’t I get any ‘likes’?’; 3) ‘Organisational learning is challenging’, including sub-themes ‘Hesitating to report’ and ‘Provides mostly superficial learning’; and 4) ‘Success inspires quality improvement project’.

**Conclusions:**

Implementing LfE mostly contributed to a positive working climate in a postanaesthesia care unit. For LfE to be worth implementing, it is essential to improve organisational learning, while minimising the negative effects of LfE, such as exclusivity issues.

**Trial registration:**

ClinicalTrials.gov NCT05794490; first registered on March 4, 2023.

**Supplementary Information:**

The online version contains supplementary material available at 10.1186/s12913-025-12626-8.

## Background

Prevailing efforts to ensure patient safety are predominantly focused on learning from errors and adverse events (Safety-I) [1]. Despite focusing on this for at least three decades, patient harm has remained a significant contributor to global mortality and disability, and it is estimated that half of these adverse events (AEs) could have been prevented [2–4]. AEs occur in 10%–24% of hospitalised patients [2, 5, 6], and this percentage is even higher (14%–30%) for surgical patients [7, 8]. Surgical-related AEs rank as the second most common type [5], with the majority transpiring during postoperative monitoring and care [7]. These persistent numbers could imply that traditional Safety-I approaches alone are insufficient to maintain patient safety [1]. It is suggested that patient safety has transitioned into the third ‘age’ of safety [9], emphasizing the need for a greater integration of Safety-II logic in complex systems to increase resilience and patient safety [1]. Safety-II involves, among other aspects, learning from how things usually go right and focusing on healthcare professionals’ well-being at work [1, 3, 9]. Learning from Excellence (LfE) is a patient safety initiative that captures episodes of ‘everyday excellence’ [10]. In doing so, LfE is arguably a system for “doing Safety-II” [10], at least partially, while improving staff morale [11]. Recognising excellent behaviour have significantly improved antimicrobial stewardship [12] and reduced cardiac arrests [13]. However, there is little empirical knowledge about how healthcare professionals can learn from everyday success in situ [14].

LfE was developed in Birmingham in 2014, with two main objectives: 1) to learn from what works well and 2) to provide positive feedback to healthcare professionals [15]. Juxtaposed to the hospital’s incident reporting system, LfE involves the voluntary reporting of a colleague’s excellent practices. The report form includes four questions: Who did something excellent; What was excellent; What can we learn from this; What can we do to make this happen more frequently. Excellence reports (LfE-reports) are then shared with the recipient, thereby providing positive feedback. To explore the events and extract valuable insights, the use of appreciative inquiry (AI) is recommended [15]. AI typically involves a conversation about an event based on positively framed questions [16]. According to Kelly et al., [15] the hypothesised effect of LfE is that it ‘would augment *learning*, enhance patient outcomes and experience through *quality improvement* work and *positively impact resilience and culture* in the workplace’ [15] p.789.

LfE is intricately connected to Safety-II and the overarching theory of resilience engineering, which defines resilience in healthcare as the capacity to “adapt to challenges and changes at different system levels, to maintain high quality care” [17] p.1. The functionality of modern healthcare organisations relies heavily on healthcare professionals’ ability to adapt, given the inherent unpredictability and complexity of contemporary healthcare. Healthcare professionals consistently compensate (work-as-done) for gaps in the underspecified ideal design for work (work-as-imagined) [1]. For instance, PACU-nurses constantly adapt to patients’ vital signs and to the perioperative team [18]. Because every patient is different, adaptations are even necessary for perioperative handoffs [19]. This transition of care and responsibility between team members is a high priority safety issue [20]. Interventions have mainly focused on standardising protocols and processes, but it is not enough simply to follow handoff checklists [19]. Understanding adaptations is crucial, with the goal of reinforcing those that lead to success and mitigating adaptability that leads to errors [1]. However, studies have found that the concept of adaptability is hard to understand, and discussing and learning from successful events poses difficulties as well [14, 21]. Therefore, there is a significant need to explore healthcare professionals’ experience in learning when using LfE, aiming to propose improvements based on current usage and thereby contributing to reality-based safety science [22].

In a postanaesthesia care unit (PACU) in a Norwegian university hospital, which is the context for this intervention study, the focus of patient safety efforts was primarily on reporting and learning from errors by using the Green Cross method [23]. Evaluations revealed a desire to extend the learning approach to successful practices as well. Consequently, the decision was made to adopt LfE. A statistically significant association exists between reduced AE rates and increased patient safety culture (PSC) scores [24, 25]. Therefore, developing a strong PSC in the PACU is important to improve surgical patient safety [3, 7, 26]. A PSC is defined as “an integrated pattern of individual and organisational behaviour, based upon shared beliefs and values that continuously seeks to minimise patient harm, which may result from the processes of care delivery” [27] p. 4. Example dimensions of PSC include Teamwork and collaboration, Learning and improvement, and Well-being [28].

Healthcare professionals’ responses to LfE have been positive, most of which is reflected in quantitative questionnaires [15, 29], mixed-method studies [30, 31], and quality improvement projects [12, 13]. The addition of qualitative methods is necessary to report contextualised factors and help understand why complex interventions fail, why they work, and how they can be optimised [32]. Qualitative methods offer rich insights into dimensions of PSC and are thus suitable for evaluating patient safety initiatives such as LfE [28]. No qualitative studies have been published on healthcare professionals’ experiences with LfE until quite recently [33]. Although the study [33] provides valuable in-depth insights, the study reports findings from five departments. PSC is a group-level property [34, 35]. Exploring and improving PSC on a single unit level is necessary. Therefore, the question remains as to how LfE was experienced by healthcare professionals in the context of a single PACU.

This study is the first of two studies that evaluate LfE within the aforementioned PACU. The statistical effect of LfE on PSC will be analysed in a further study. Together they capture a richness of data from one setting.

### Aim and research questions

This study aimed to describe healthcare professionals’ experiences with Learning from Excellence (LfE) six months after its implementation in a postanaesthesia care unit.

The following are the specific research questions based on the hypothesised effect of LfE [15]:

1. How do healthcare professionals experience LfE as a strategy for improving well-being?
2. How do healthcare professionals experience LfE as a strategy for learning?
3. How do healthcare professionals experience LfE as a strategy for quality improvement?

## Methods

### Design

To obtain a deep understanding of the healthcare professional’s experience with LfE, a qualitative descriptive design with focus group (FG) interviews was implemented. The Consolidated Criteria for Reporting Qualitative Research provided a useful framework for reporting [36] (Supplementary Material 1).

### Setting

The LfE intervention was carried out in a 25-bed general PACU in a Norwegian university hospital. The PACU ran 24 h a day, 7 days a week, and admitted approximately 1000 patients per month, ranging from infant to geriatric. The PACU provided overnight care for complex patients and intermittently cared for intensive care unit patients as an overflow area. It provided non-invasive and invasive mechanical ventilation and active management of cardiovascular physiology, including vasoactive drugs. The number of PACU staff during the intervention period was 76 nurses (*M*_*age*_ = 44 years; 12% male and 88% female), 2 nurse managers and 2 certified nursing assistants. Two chief anaesthesiologists worked in the PACU during the daytime on weekdays. The anaesthesiologists’ unit that serves the PACU consisted of 45 anaesthesiologists and 27 residents. Residents worked in the PACU for three months as part of their training to become anaesthesiologists. Most of the anaesthesiologists work in the PACU regularly in daytime during pre- or postoperative follow-up on patients or during evening and night shifts.

### Participants and recruitment

A convenience sample of healthcare professionals, including nurses, anaesthesiologists and residents who met the inclusion criteria, was conducted. This included all nurses (*n* = 76) and nurse managers (*n* = 2) who had been working in the PACU for at least six months and all anaesthesiologists (*n* = 45) and residents (*n* = 27) who had some experience with LfE. Invitations were sent out via email from their respective nurse and physician managers. In addition, invitations were communicated via posters in the PACU, and announcements were made twice during morning and afternoon shifts by GB. Nine anaesthesiologists and 22 nurses volunteered. In all, 24 healthcare professionals participated in the FG interviews, of whom most were nurses (*n* = 17), in addition to physicians (*n* = 7). See Table 1 for the participants’ characteristics.

**Table 1:** Participant characteristics (*n* = 24)

**Characteristics**		**n**	**%**
Gender	Female	19	79
	Male	5	21
Profession	RN^a^	4	17
	CCN^b^	10	42
	CNS^c^	1	4
	Nurse manager	2	8
	Anaesthesiologist or residents (physicians)	7	29
Age (in years)	23–29	4	17
	30–39	7	29
	40–49	5	21
	50–59	7	29
	60–69	1	4
Years of experience in healthcare	1–10	9	37.5
	11–20	3	12.5
	21–	12	50
Years of experience in the anaesthesia department	1–10	18	75
	11–20	3	12.5
	21–	3	12.5
Members of the LfE^d^ team		5	

^a^Registered Nurse (Bachelor of Science)

^b^Critical Care Nurse (Postgraduate registered nurse/Master of Science in nursing)

^c^Clinical Nurse Specialist responsible for teaching

^d^Learning from Excellence

### Study intervention

**Fig. 1:**
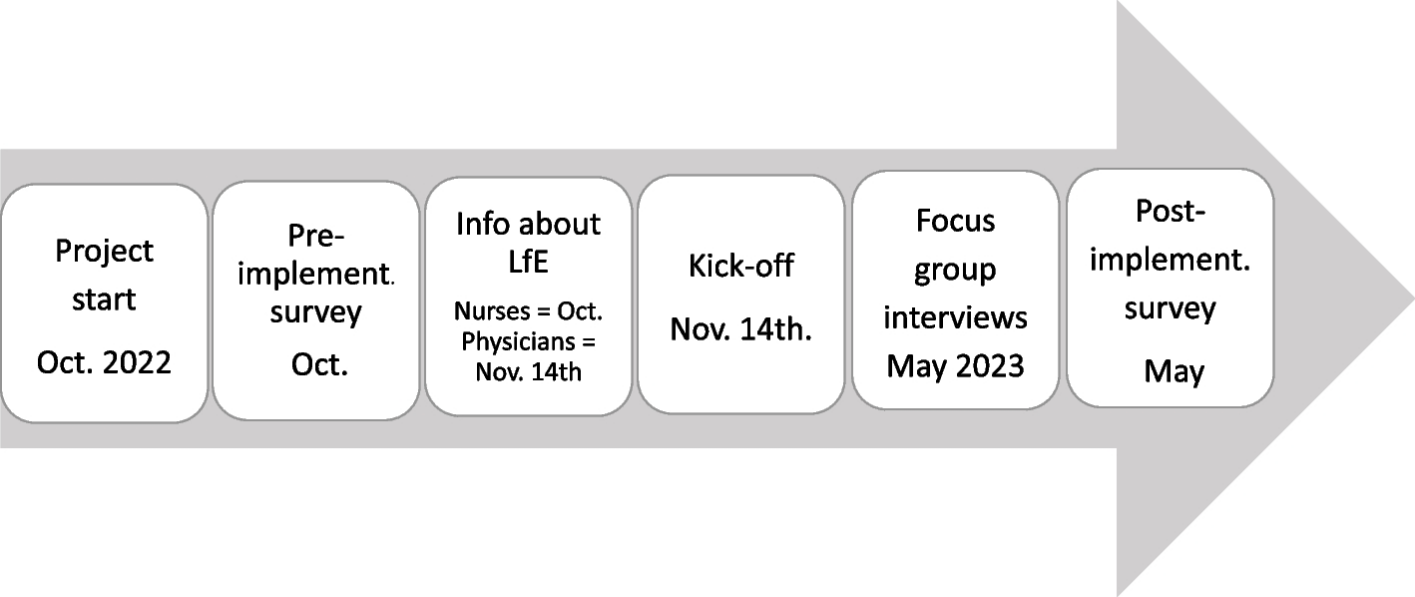
The progress plan for the implementation

The decision to implement LfE was initiated by the PACU management. According to the progress plan (Fig. 1), the LfE team was responsible for planning and implementing a modified version of LfE (Table 2), including mini-AI (Supplementary Material 2). The LfE team consisted of a nurse manager who was the project leader, two nurses, two anaesthesiologists, an IT consultant, a patient representative and a PhD student who served as an external adviser (GB).

**Table 2:** A modified version of the step-by-step working process of LfE [37]

What is done:			How:
**Reporting**	1.	Voluntarily reporting of excellence by QR^b^ codes displayed on posters at the premises using mobile phone (Initially, only the first two questions were included based on the assumption that praising a colleague was a valid goal alone, but after a few weeks, all four questions were included.)	No restrictions or guides on which types of episodes to report. Four main questions: 1. Who did something excellent? 2. What was excellent? 3. What can we learn from this? 4. What can we do to make this happen more frequently? • I work here (name voluntarily) • I am a patient/next of kin (no name allowed)
**Feedback**	2.	Reporters receive an automated text message of acknowledgement:	‘Thank you for assigning this report to a colleague. The person in question will receive information of what you found excellent, and the episode will be used in work on improvement’
	3.	Recipients of an LfE^a^ report receive a text message notification from the LfE^a^ team, specifically from the nurse manager and an anaesthesiologist, for nurses and anaesthesiologists, respectively, stating:	‘Hello [name]! You have received an LfE report from [name]. [Insert text from original report] Excellent work! Sincerely, [name]’
**Learn**	4.	A one-hour meeting is held every other week for the LfE^a^ team, consisting of interprofessional healthcare professionals, where: a) the LfE^a^ team decides on two reports to explore in depth, based on reading/reviewing/discussing the latest reports. Subsequently, a nurse initiates an informal mini-AI^c^ (Additional file 2)	AI^c^ is either a group dialogue between those submitting and receiving an LfE^a^ report or an individual interview with either one of them if the former is not possible. Duration ca. 5 min. Using these questions: • **Definition:** Purpose of meeting Congratulation (acknowledging) • **Discovery**: Exploring what was excellent in this episode • **Dream:** What needs to be done to make this happen more often? (measures) • **Design:** How can we promote and share this excellent practice?
		b) ^*^Information from the AI^c^ interview becomes the ‘LfE^a^ of the week’	Information from the event is written in a book and is read aloud before each shift for a week, for PACU staff for inspiration and learning. The name of the recipient was disclosed at the request of the nurses, who found that by omitting the name of the recipient, the event became unrelatable
		c) Original text from one or two LfE^a^ reports per week is displayed on an information screen in the PACU^d^ break room	Original text is displayed with illustrating lively pictures for inspiration and learning
	5.	LfE^a^ reports are summed up monthly by the LfE^a^ team. A summary is displayed on a whiteboard in the hallway for everyone to see:	Number of reports (statistics) is displayed. Anonymised text from original LfE^a^ reports is displayed in the following categories: professional work/innovation/initiative/communication/management/good teamwork
	6.	One LfE^a^ report, chosen by the LfE^a^ team, becomes a focus point for work on improvement	Measures are taken using PDSA^e^ to improve work: Mandatory pre-round meetings regarding all intensive care and intermediate patients

^a^Learning from Excellence

^b^Quick response

^c^Appreciative inquiry

^d^Postanaesthesia care unit

^e^Plan-Do-Study-Act-cycles

^*^Not included for the anaesthesiologists

**Fig. 2:**
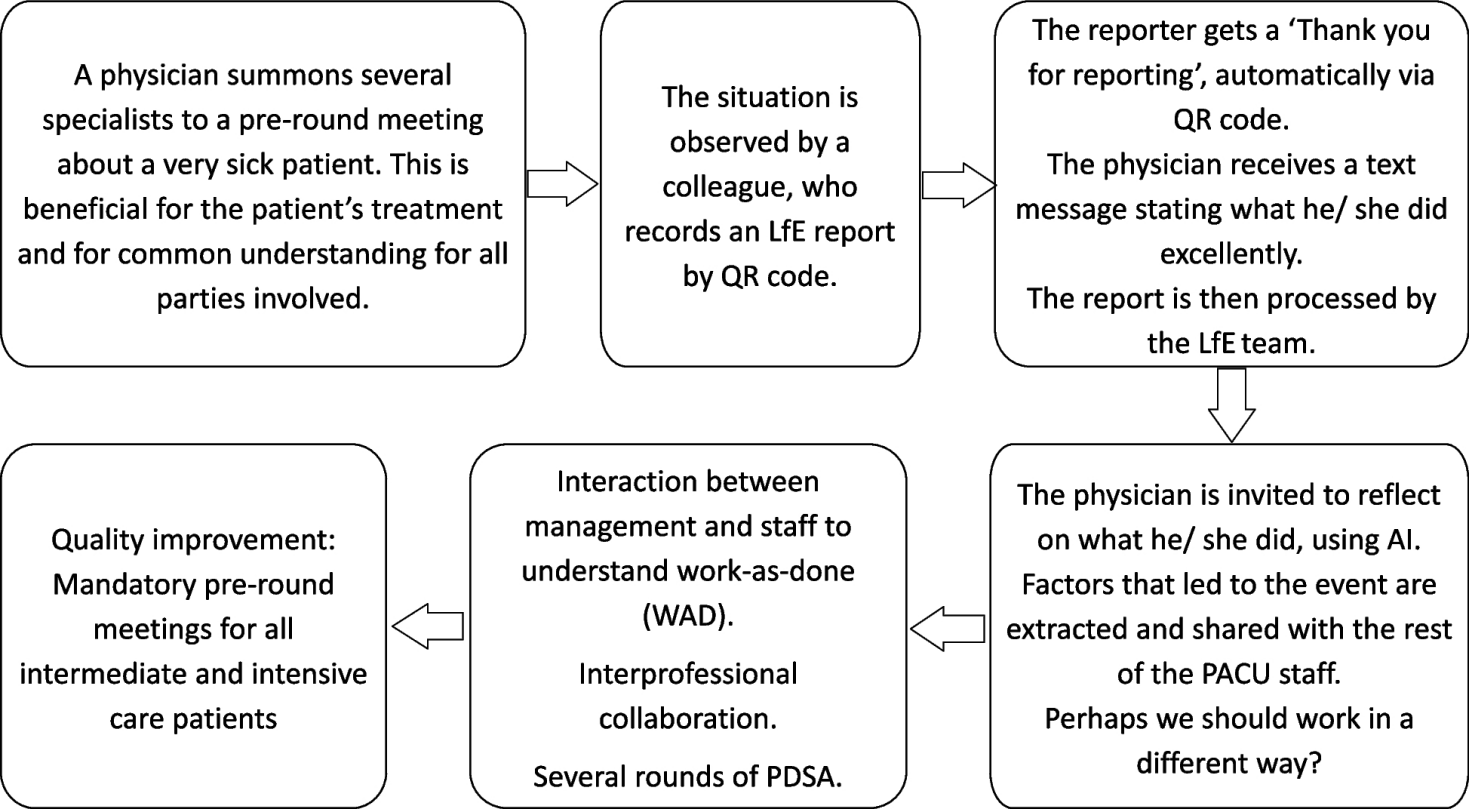
Example of the reporting and learning process of the modified LfE^.^ AI = Appreciative inquiry; LfE = Learning from excellence; PACU = Postanaesthesia care unit; PDSA = Plan-Do-Study-Act cycles. QR = Quick response

Kotter’s [38] eight-step process for leading change guided the implementation (Table 3). An example of how LfE was used is illustrated in Fig. 2. Whereas PACU nurses were introduced to LfE as a patient safety initiative to learn from successful events *and* increase positive feedback, anaesthesiologists were introduced to LfE solely as an initiative to increase positive feedback. This was based on assumptions about what was feasible within the anaesthesiologist’ unit at that time. It was perceived feasible to increase positive feedback, but not to disseminate examples of everyday excellence for organisational learning.

**Table 3:** Implementing LfE according to Kotter’s [38] eight-step process for leading change

**PREPARATION**		
** 1 Need for change**		- The interprofessional frontline clinical staff wanted to increase positive feedback and focus more on successful events - The LfE^a^ patient safety initiative was deemed eligible as a tool to increase positive feedback and facilitate learning from success - A pre-implementation survey was conducted before implementation for PACU staff
** 2 Establishing a LfE**^**a**^** team**		- Consisting of a nurse manager, two anaesthesiologists, two RNs ^b^, a patient representative, an IT consultant and an external adviser (GB) (*n* = 8) - The LfE^a^ team met weekly before and every two weeks after the implementation
** 3 Establishing a vision and a strategy**		- The LfE^a^ method according to www.learningfromexcellence.com was adjusted to fit the PACU^c^. A vision to increase reporting and learning from excellence was agreed upon. This included reports from staff and patients/next of kin - A project plan was conducted consisting of goals and a plan for implementation and evaluation
**ACTION**		
** 4 Conveying vision to the staff**		- Written information about the LfE^a^ method was given to PACU^c^ staff through emails and via information screen, describing the LfE^a^ method’s ‘why, when and how’ - Oral information to the PACU^c^ staff and anaesthesiologists regarding the LfE^a^ method was provided via a 15-min presentation by members of the LfE^a^ team - PACU^c^ staff received oral information through a staff meeting
** 5 Empowering**		- A whiteboard in the hallway and a digital information screen were used to display information - QR^d^ codes were displayed on posters in the PACU^c^ and in the anaesthesiologists’ meeting room
** 6 Small wins**		- The kick-off was celebrated -The first 100 reports were celebrated with cake in January 2023 - Work on improving pre-round meetings was displayed via the weekly PACU staff email and on the whiteboard in the hallway - 100% achieved pre-round meetings were celebrated with cake in April 2023
** 7 The LfE team did not stop the daily implementation**		- The LfE^a^ team met for status and problem-solving - The project leader and one of the anaesthesiologists forwarded LfE^a^ reports to the recipients daily for PACU^c^ staff and anaesthesiologists, respectively - One nurse initiated mini appreciative inquiry regularly, thereby exploring one LfE^a^ report per week, which became the ‘LfE of the week’ - Episodes of good practice were displayed on posters, on a digital info screen, in the weekly PACU staff email and through daily readings of the ‘LfE of the week’
**CULTURE**		
** 8 The GC method was culturally rooted**		- The implementation continued. This takes years to implement and requires daily, monthly and annual follow-ups - Evaluation through Annual Norwegian hospital survey ‘ForBedring’ (March), post-implementation survey (May) and focus group interviews (May–June) - Autumn 2023: planning monthly meeting spaces for PACU^c^ staff where successful events will be shared and learned from

^a^Learning from excellence

^b^Registered nurse

^c^Postanaesthesia care unit

^d^Quick response

### Data collection

Data collection based on exploratory FG interviews with a semi-structured interview guide was deemed appropriate for this study [39]. The interview guide was pilot-tested with three nurses familiar with LfE; however, they did not meet the inclusion criteria. One follow-up question was added after the pilot: How do you think LfE could be further developed? The interview guide resulted in six open-ended questions (Siupplementary Material 3).

From May to June 2023, six semi-structured FG interviews were conducted. The FG interviews took place in rooms adjacent to the PACU during working hours with three to five informants. The FG interviews were performed as a dialogue between the informants [39]; their mean time was 1 h and 3 min (52 min–1 h and 26 min).

FG interviews allow informants to be actively encouraged in discussions, thus generating diverse views and a rich account of healthcare professionals’ experiences [39]. The nurses’ FG interviews (*n* = 3), and the physicians’ and LfE team’s FG interviews (*n* = 3) were moderated by GB and ETD, respectively. GB (female critical care nurse, PhD student) had previously worked in the PACU and had helped implement the Green Cross method and LfE. GB was therefore familiar with some of the participants. This was her fourth FG interview study. ETD (female MD, Internist, PhD, Senior researcher) had not worked in the PACU. She was familiar with one of the participants from previous work, but not in-depth. This was her first interview study. The moderator presented an introduction to the study. The observer asked follow-up questions if necessary. The observer took field notes and read aloud a summary, which was confirmed by the informants at the end of the FG interview. The researchers discussed this note for increased understanding and reflexivity [40]. All FG interviews were audiotaped, transcribed verbatim and de-identified [40]. Five FG interviews seemed feasible, but an additional FG interview was held to cover significant variations [40].

### Data analysis

A reflexive thematic analysis, as described by Braun and Clarke [41], was deemed appropriate for this study, as it is a paradigmatic qualitative approach to interpreting qualitative data and facilitates the generation of themes in a data set. In reflexive thematic analysis there is no single ‘correct’ interpretation, and subjectivity and creativity is emphasised as an analytic resource [42]. The authors’ clinical experience and theoretical knowledge about Safety-I and Safety-II was used to understand, interpret and discuss the data [43]. While assuming that the authors’ theoretical assumptions influence data interpretation, it is recommended that researchers specify these assumptions [43]. The thematic analysis was underpinned by a constructivist epistemology that views reality as subjective, and data from FG interviews as the co-creation of the participants and the researchers [44]. A predominantly inductive approach of reflexive thematic analysis was performed, meaning data was open-coded and the informants’ meanings were emphasised [40]. Initially, and in line with Braun and Clarke [42], GB performed the data analysis individually and subsequently with the whole research team (Table 4). Collaboration among all the authors enhanced interpretation and understanding [40].

**Table 4:** How the analytical process according to Braun and Clarke [40] was applied

**1. Familiarising**	The authors read and re-read the data to become familiar with its content. GB took notes on her analytical observations and insights, both in relation to each transcript and to the entire dataset. This was discussed with all authors for the purpose of reflexivity. ‘What is this about?’
**2. Coding**	Each transcript was put in a separate Excel spreadsheet. GB created brief code labels in a column alongside the transcript that captured important features that were relevant to the research question. The codes could be semantic (explicit) or latent (implicit) and were refined and added to as transcripts were revisited over time. The Excel spreadsheet made it easy to ensure that areas were not neglected. Three separate rounds of coding were conducted. Then, all codes were organised together for later stages of analysis
**3. Generating initial themes**	Larger patterns across the dataset were looked for, and the codes were grouped into possible themes in a separate Excel spreadsheet. GB tried not to have her coding and themes steered by ideas from previous research to allow for an inductive approach
**4. Theme development and review**	In this phase, the themes needed a considerable redevelopment based on the following five criteria: Is it a theme or a code? Does the theme tell me something useful about the data and the research question? What does the theme include/exclude? Are there enough data to support this theme? Are the data too diverse and wide-ranging? Theme and sub-theme maps were drawn on paper that opened up for creativity and to visualise the relationships between the themes/sub-themes and between the research questions and the themes. This was discussed with all authors for the purpose of reflexivity
**5. Refining, defining and naming themes**	A definition of each theme was written, focusing on the central organising concept of the theme, the boundaries of the theme, and what the theme contributed to the overall analysis. The content within a theme could not be contradictory, for example both good and bad experiences
**6. Writing up**	An illustrative treatment of data extracts was used in reporting the themes

## Results

This study reports four themes regarding nurses’ and physicians’ experience of LfE six months after its implementation in a PACU. These themes were termed as follows: ‘Facilitates a more positive working climate’, ‘Why don’t I get any ‘likes’?’, ‘Organisational learning is challenging’ and ‘Success inspires quality improvement project’. The theme characteristics are displayed in Table 5. Quotations are numbered by FG and informant numbers, respectively. Words emphasised by the informants have been marked in bold.

**Table 5:** Summary of characteristics for the four themes, and their relevance to the research questions

Research questions	Themes	Subthemes	Characteristics
1. How do healthcare professionals experience LfE as a strategy for improving well-being?	**Facilitates a more positive working climate**	***Helps spread positive feedback***	Displaying good practice via multiple channels helped healthcare professionals gain a more positive perspective
		***Feeling seen and motivated***	Recipients of LfE reports experience positive emotions that affect their work life
	**Why don’t I get any ‘likes’?**		Concerns for those who do not receive an LfE report, thinking this may lead to worries of inferiority. The focus is on the person rather than the event and the system
2. How do healthcare professionals experience LfE as a strategy for learning?	**Organisational learning is challenging**	***Hesitating to report***	Obstacles, such as excessive questions in the reporting form and a preference for verbal feedback, made reporting strenuous
		***Provides mostly superficial learning***	Too little analysis of the successful event in a system perspective to change routines and practice
3. How do healthcare professionals experience LfE as a strategy for quality improvement?	**Success inspires quality improvement project**		Doing more of something positive is inspiring

### Facilitates a more positive working climate

The display of good practice helped healthcare professionals focus on excellent work. The threshold was lowered for giving verbal feedback to each other. Positive feedback felt great and helped motivate them. This is described in the two sub-themes *Helps spread positive feedback* and *Feeling seen and motivated*.

#### Helps spread positive feedback

The managers thought it was important for employees to give positive and constructive feedback to each other. They found that LfE was a tool to help this cause, as illustrated by a nurse (1:1):

LfE has contributed to us promoting the positive. Because we humans are not very good at saying positive things to each other […] one forgets it […] but after LfE, it becomes in focus, and from there it’s also easier to notice the positive things, and easier to send an LfE-report […] but without any tools like that, then I sort of feel that we humans aren’t very good at that.

The focus on good practice displayed across the PACU lowered the threshold for giving specific compliments to each other. Healthcare professionals stopped taking help from colleagues for granted and thanked each other more, even for small things. This did not go unnoticed by the patients who commented on the good atmosphere in the PACU (3:12): ‘*I hear patient say Oh! It is so nice to hear that you are being so kind to each other*’. Some nurses assumed that the positive working climate made the patients feel safe and well taken care of and that increased positive feedback could improve teamwork and help maintain the PACU as a popular place to work.

The LfE quick response code made it easy to give positive feedback to colleagues from other units who left before one had the chance to give feedback verbally. The healthcare professionals thought it was nice to have a system that facilitated positive feedback across the whole hospital. Being able to report anonymously presumably made it less embarrassing to give positive feedback to higher-ranking colleagues (e.g. nurses to physicians, residents to anaesthesiologists).

Hearing the ‘LfE of the week’ being read out balanced the focus on errors via the Green Cross method, thus creating a more positive working climate, as disclosed by a nurse (2:6):

I was so weighed down by the Green Cross method […] reading out ten things in the morning that we had done wrong during the evening shift […] I can´t bear to listen to it. […] and now we finish off by reading out what went well. It is wonderful! […] I have missed it so much. [...] Actually, I have given this a lot of thought.

#### Feeling seen and motivated

One of the main goals of implementing LfE was to acknowledge healthcare professionals, with the purpose of lifting them up and increasing job satisfaction. The healthcare professionals expressed that receiving an LfE-report was pleasant and meaningful and made them feel happy and satisfied with themselves and the job. Moreover, it was energising and motivated them to put in extra effort into their patients. The acknowledgements were important at a deeply human level, as described by a physician (6:24): ‘*Of course it’s very good to feel seen, it’s fundamentally good I think, for everyone*’. The fact that someone chose to single you out and compliment something you did well felt like being seen for the person you are, not just ‘another white coat’, and this was highly valued in the high-tech environment of the PACU.

The LfE-report was a text message they could look back on as a ‘concrete proof’ of a job well done, thus being remembered months after it was received. It was more specific than the usual ‘well done’ and could be particularly useful to new employees. It gave them a sense of being valued team members, increasing their self-confidence, as described by a resident (6:22):

It reassures me that what I do is correct […] that my colleagues think I do a good job. When I started in anaesthesiology, it was like this: If you don´t hear anything, it means it’s OK […] you only hear it when you have messed up. And it´s like… you fumble a bit and do not quite know if you are doing a good job or not […] And this can be helped by LfE or by just giving more positive feedback, which is somewhat lacking in our education to become an anaesthesiologist.

### Why don’t I get any ‘likes’?

As LfE is a subjective and not an objective tool, healthcare professionals were assumed to give LfE-reports based on factors other than colleagues’ objective skills. This could create an unfair and brutal system. Some were assumed to receive more LfE-reports based on their popularity, social skills and positive energy. The modest ones were assumed to receive fewer LfE-reports, if any. Moreover, experienced nurses were less likely to receive an LfE-report than more inexperienced nurses who had recently learned a new skill. Although the more inexperienced needed more positive feedback to help them grow professionally, being overlooked fuelled slight dissatisfaction. Some nurses specifically wanted LfE-reports from physicians. Discreet comments in the corridor were heard from both nurses and physicians: (5:21): ‘*I never get an LfE-report. I do a great job, I go home tired every day, but I never get one*’.

You could easily imagine that you were the only one who did not receive any LfE-reports, whereas everyone else did. The healthcare professionals worried that those who did not receive LfE-reports could feel sad, excluded and not appreciated, and that this could be harmful. Some hesitated to report excellent events for this reason. As illustrated in a discussion between three physicians:

It becomes a social media-effect. You think everyone else gets LfE-reports. (5:18)

Yes! That’s exactly what I think. (5:21)

This is ‘likes’. It’s just another way to give a ‘like’. And it’s relentless, isn’t it? (5:20)

The ‘LfE of the week’ was read aloud for inspiration and learning purposes and included the recipient’s name. The LfE team made a deliberate effort to highlight the work of quieter individuals, demonstrating that LfE-reports were not exclusive to extroverted colleagues. However, revealing names caused listeners to focus on the person rather than the event, as stated by a nurse (2:6):

I was surprised of my feelings […] for each new name read aloud, it was like… I don’t feel envy or something like that […] but you feel it. It becomes very personal instead of system-focused […] even though it is super-nice to know which nurse did it, I get very focused on the nurse more than the event. For some reason.

The healthcare professionals stated that the recipients certainly deserved to be recognised. However, at the same time, they too deserved to be acknowledged, as stated by a nurse (2:7): ‘*What about me then? I worked overtime to help out with that really sick patient*’. The healthcare professionals concluded that LfE was too good to stop completely but that the LfE team should be very mindful of those who did not receive any LfE-reports.

### Organisational learning is challenging

The participants in this study found reporting to be arduous, and several did not learn much from others’ successful events. This is described in two sub-themes: *Hesitating to report* and *Provides mostly superficial learning.*

#### Hesitating to report

Initially, the LfE-report form contained two questions: ‘who, and what was excellent’. However, after a few weeks, two questions were added to emphasise the learning focus of the successful event, according to LfE: ‘what can we learn from this’, and ‘how can we make it happen more frequently’. The additional questions made the reporting strenuous, as explained by a nurse (1:2):

At the beginning I wrote kind of simple reports but as time went on, it seemed like bigger demands were placed on us […] you had to analyse it and evaluate it […] then it is not easy to report, you have to plan for it.

It was difficult to put into words what could be learned from an episode. Oftentimes, they just repeated the wording, illustrated by a nurse (1:5): ‘*What the nurse does really well is to facilitate good flow in the PACU […] and what we can learn from this is to facilitate good flow in the PACU [laughs]*’. The healthcare professionals complained that the added questions were too reminiscent of the arduous incident reporting system. They had patients to attend to and did not have time to think deeply about further implications, as stressed by a nurse (1:1):

It is such a burden on me, why should I take a responsible part in figuring out what can be done to make this happen more frequently? That is up to you [LfE team] to figure out.

Healthcare professionals were further hampered by not knowing what qualified as excellence. Even though ‘everything’ could be reported, they supposed that it should be about something extraordinary. Things they were expected to do did not qualify as excellence, as explained by a nurse (4:17):

We expect people to be good at their job and to do their job […] I expect my colleagues to notice when a patient gets sicker, it is not a WOW when someone picks up on that. Hundreds of colleagues have done that before without getting an LfE-report […] that´s our job […] that’s the reason the patients are at the PACU.

Instead of reporting, it was easier to give compliments to each other face-to-face. The healthcare professionals preferred this over the often-anonymous LfE-reports, as verbal praise was seen as nicer and more significant for personal growth.

The physicians first realised that the LfE-reports were read by the LfE team and sometimes a manager after a considerable amount of time. Feeling deceived, this further reduced their willingness to report.

#### Provides mostly superficial learning

The disinclination to report hindered organisational learning about conditions that promote safe care. A nurse exemplified an event that, had it been reported and shared, could have increased patient safety (4:15):

I had an experience at a nightshift where I did an excellent job. I kept the patient [overnight] at the PACU, despite the inconvenience this caused. The patient had to be sent back to the operating room […] This was really something that others could learn from [...] the observations that I always do, that no one else does.

Selected LfE-reports were explored using mini-AI to extract learning. It was challenging to find time for an informal interview with the reporter and the recipient, as they frequently worked different shifts and had patients to attend to. Therefore, mini-AI took about 5 min. The healthcare professionals who attended the mini-AI found it useful, even if it did not generate new information. The ‘LfE of the week’ was found to be too superficial to learn from; e.g. hearing that some communicated well did not make others better at communicating. The nurses wanted to know more about the specifics, for example whether ‘closed loop’^1^ had been used. They did not want to hear about the same event for a whole week; moreover, they suggested that only the most important events were reported back to staff. However, hearing the ‘LfE of the week’ could make them reflect on how they would act in the same situation, as stated by a nurse (4:16):

It's something about the fact that everyone hears the same thing at the same time, so that there is an opening to talk about it if it is something special.

The nurses requested formal meetings to discuss successful work, as they had no place to discuss successful events other than in rare debriefings. The healthcare professionals found it rewarding to reflect on excellence in the research FG interviews. By reflecting together, their understanding of the purpose of LfE was deepened.

### Success inspires quality improvement project

Reporting and studying successful work rather than taking it for granted was a driver for quality improvement work. During the implementation of LfE, an improvement project was initiated, as explained by a nurse (1:2):

Something that I think has been really positive that has come out of LfE, is that we now conduct pre-round meetings […] We […] have been working to achieve this for a long time […] we wanted mandatory pre-round meetings for the sickest patients. But we haven’t quite got there. But now […] the management also worked to achieve this.

Two LfE-reports described a successful pre-round meeting, including several specialists, and how this was beneficial for the patient and all parties involved. Mandatory pre-round meetings in the physicians’ office with the nurse and the physician were a project that everybody supported. A structured Plan-Do-Study-Act was performed, as explained by a nurse (3.10):

We decided together with the physicians that we wanted this for all relevant patients, not just arbitrary ones […] then we started counting for four weeks, and everyone was involved […] it was an interprofessional collaboration […] this has benefited, mostly the patient, but also the work of the nurses and the physicians, it was very successful.

A common goal and interprofessional collaboration were pointed out as important factors contributing to the success of this initiative. Some said that learning from success was more motivational than learning from errors, as explained by a nurse (4:14): ‘*It’s much nicer to work to get better at something positive than to stop doing something wrong*’. Everyone agreed that this improvement project had increased patient safety, as it reduced multitasking and allowed them to focus deeply on one patient at a time.

## Discussion

**Fig. 3:**
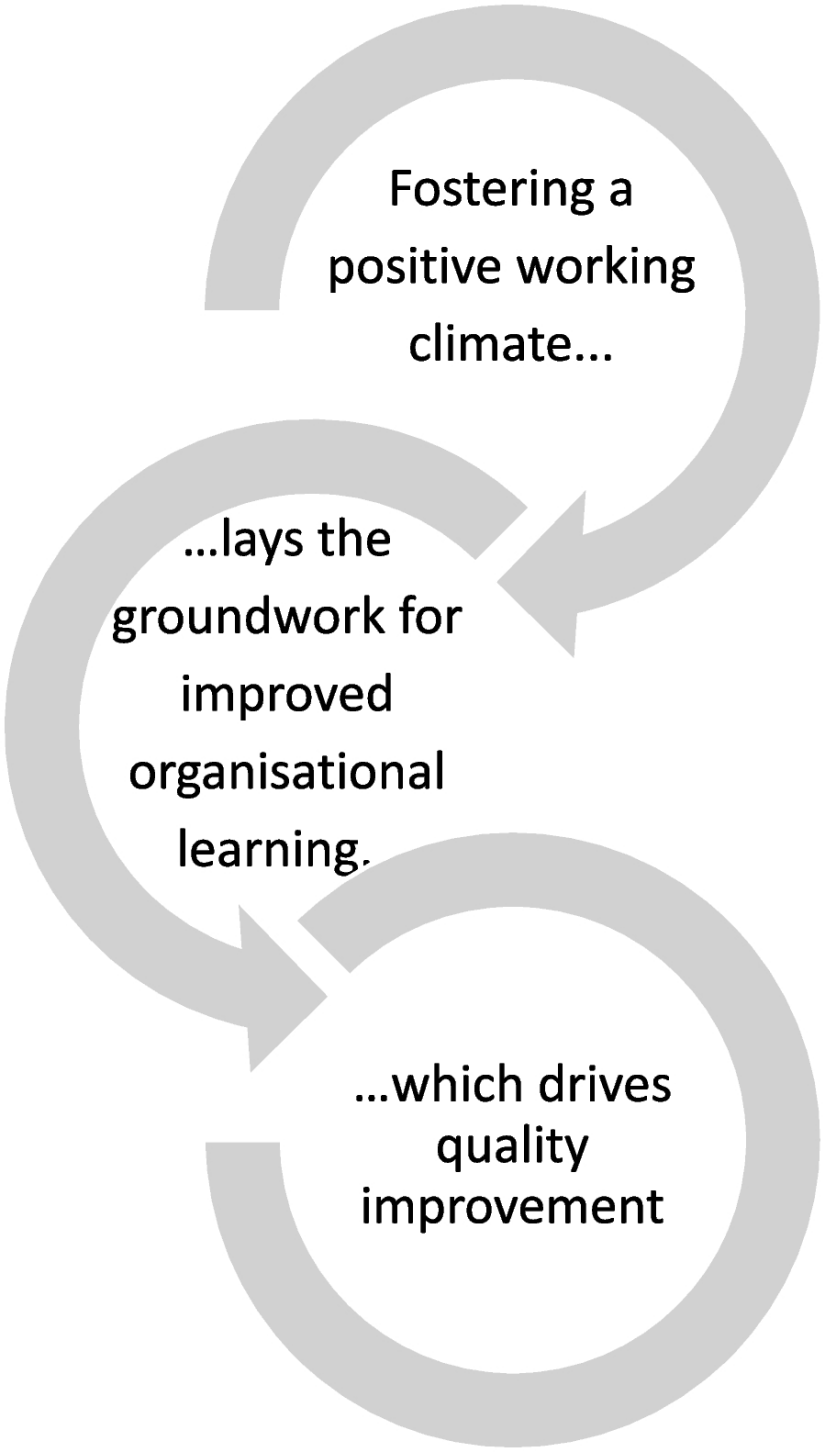
The relationship between the themes

This study aimed to describe healthcare professionals’ experiences with Learning from Excellence (LfE) six months after its implementation in a postanaesthesia care unit (PACU). Hypothetically, reporting and studying excellent work rather than taking it for granted may foster a positive working climate and lay the groundwork for improved organisational learning, which then drives quality improvement work (Fig. 3). The four themes developed from the FG interviews indicate that the intervention contributed to a positive working climate, although the healthcare professionals were concerned about those who did not receive LfE-reports. Moreover, reporting and organisational learning were challenging, although LfE led to a successful quality improvement. The following discussion is organised according to the three research questions.

### How do healthcare professionals experience LfE as a strategy for improving well-being?

From the healthcare professionals’ perspective, the most important aspect of LfE seemed to be that it helped them notice the successful work being done every day, thus improving their well-being. This is illustrated in the theme *Facilitates a more positive working climate*. Due to the prevailing negativity bias, positive events are taken for granted, and an effort must be made to focus on successful events throughout the day [45]. By displaying successful work, it was easier to remember to thank each other more. Expressing gratitude cultivates social bonds for both the expresser and the recipient [46]. This improves relationships and psychological safety, which is key to building healthy teams [46, 47]. In particular, positive feedback from senior physicians was sought by nurses and residents.

Positive feedback made the healthcare professionals feel happy, seen and motivated. This agrees with a recent LfE study [33]. This is linked to the Well-being dimension of PSC [28]. Positive feedback enhances performance and increases self-efficacy—the belief in ones’ own abilities to tackle future tasks [48]. Self-efficacy is a well-known psychological resource that buffers the negative effects of stress [48]. Together with joy at work, this stands in stark contrast to clinician burnout [48, 49]. Because burnout has reached epidemic proportions among healthcare professionals, interventions such as LfE should therefore be welcomed [49]. Being seen as a person and not just another ‘white coat’ was especially important in the busy, high-tech environment of the PACU. A good local team climate is critical for high-quality patient care delivery [50, 51]. The patients commented on the kindness between colleagues in the PACU, which was also extended to them. Kindness is linked to improved patient outcomes, better staff experience and retention, and better teamworking scores [52]. Kindness should therefore be a central starting point for how healthcare is delivered, which is something LfE seems to facilitate [52].

While the finding suggests that the PSC factor ‘well-being’ could be increased by recognizing excellent work [28], this was disputed by the exclusivity issue illustrated in the theme *Why don’t I get any ‘likes’?* The participants experienced that both anonymous and non-anonymous LfE reports could make one imagine that they were the only one who did not receive LfE-reports. This may have a detrimental effect on well-being and psychological safety. That is, the perception of whether it is safe to be candid and speak up in the team without fear of negative consequences [53]. Over time, this can negatively affect team morale and reduce patient safety [47, 53, 54]. Interestingly, jealousy and the risk of leaving someone out was also experienced in three previous LfE studies [30, 31, 33]. People felt resentful when they did not get ‘awards’, and cliques of friends reported each other [31, 33]. ‘Popularity bias’ and uneven acknowledgement through LfE-reports may not be easy to avoid. The participants in present study suggested that the LfE team should be very mindful of those who did not receive any LfE-reports. This stresses the managers’ role in fostering a culture that values diverse contributions and in building psychological safety. To this end, Human Factors tools such as e.g. the Diversity Icebreaker®, which aims to improve the communication and interaction in the group, have been developed and can be used [55]. Managers may also initiate open discussions about possible uneven acknowledgement through LfE-reports with staff to prevent toxic positivity [56]. Managers may use positive leadership walk-rounds where they ask individual staff about what is going well in addition to what could be better [57]. This is associated with ‘well-being’ and improved PSC [57]. Encouraging more verbal positive feedback between staff may also mitigate uneven acknowledgement through LfE-reports. Participants in another study suggested that raising awareness of the system could reduce the risk of feeling left out [33].

### How do healthcare professionals experience LfE as a strategy for learning?

As illustrated in the theme *Why don’t I get any ‘likes’?*, lack of trust in LfE was a barrier to reporting and learning. Fostering a positive working climate, which includes psychological safety, lays the foundation for improved organisational learning [53]. However, that demands that the events are analysed in a system perspective. On the contrary, in this study, the healthcare professionals reasoned that, since LfE-reports are based on subjective sentiments, with a focus on individuals, they would favour popular colleagues and be harmful to those who never received any. This made some hesitant to give LfE-reports. Moreover, a prerequisite for trust is knowing who manages the LfE-reports. This was under-communicated to the physicians. To increase trust, it is important to have a shared understanding of LfE’s purpose [30], i.e. learning from successful adaptations in the complex healthcare system, to increase patient safety [1, 15]. This is a new approach to patient safety and is not easy to grasp [14, 21]. Clear and consistent information about LfE may be the most important learning needs of staff prior to implementing LfE [30]. That is, what it *is* and what it *is not* [11]. Without a clear vision, the implementation is unlikely to succeed [38]. Another barrier to reporting was the arduous reporting form, as illustrated in the theme *Organisational learning is challenging*. One way to make reporting easier may be to omit the question ‘How can we make this happen more frequently?’, which in this study did not yield much information.

Verbal, individual, in-the-moment feedback increased after the intervention and was considered more meaningful for personal growth than written LfE-reports. For this reason, the participants desired more positive feedback–especially from their superiors. For instance, positive verbal feedback was somewhat lacking in the education to become anaesthesiologists, where no feedback meant you did an’OK’ job (‘Feeling seen and motivated’). The desire for more positive feedback agrees with a previous study from the same PACU [23]. This relates to the Learning dimension of PSC [28], and suggests that both verbal and written positive feedback is needed to improve the learning culture. The participants’ sentiments are consistent with a large synthesis of meta-analyses by Hattie [58]. It concluded that verbal feedback is one of the most powerful influences on students’ achievement, even more effective than students’ prior cognitive ability [58]. As stated previously, positive feedback can increase self-efficacy and enhance performance [48]. It is therefore not surprising that positive verbal feedback contributed to surgical trainees’ learning progression and increased their motivation and performance [59]. It also improved their well-being and confidence [59]. Future implementers should therefore encourage positive verbal feedback between healthcare professionals, not only written feedback. One way to do so is by systematically including positive feedback into team-huddles, one-on-one check-ins, or mentorship programs for different groups of healthcare professionals. Reflection and learning from successful events may be best conducted directly after a procedure or interaction while it is still remembered. The focus of such educational feedback is ‘what am I good at’ [60]. For example, good at helping others to do their job in a better way. However, as described in the subtheme ‘Helps spread positive feedback’, saying positive things to each other is easily forgotten. Thus, a culture change seems necessary, where verbal positive feedback is seen as the most important part of a good learning environment, along with direct instructions [58]. The participants in present study experienced that LfE was necessary to notice and promote the positive and can thus be seen as a tool for changing the culture towards a more positive view of patient safety and teamwork. In addition, visual aid such as pocket guides for mentors could be useful to ensure consistency.

Written LfE-reports were excellent alternatives when verbal feedback was not practicable, especially for colleagues working in other units. These LfE-reports were remembered months after being received, thus increasing their impact. This agrees with a recent study [33]. Ensuring that all team members feel valued, written or verbal, is one way to increase psychological safety in a team [61]. Psychological safety is a property of the Teamwork and collaboration dimension of the PSC, and it is crucial for units to be able to learn and implement changes [47, 54].

Posters and ‘LfE of the week’ were considered inspiring but too superficial to learn from. The healthcare professionals in this study wanted formal reflections based on LfE-reports. Problems with learning was also found in a recent British LfE-study [33]. Their experiences are supported by learning theory [14, 62]. Organisational learning necessitates 1) *representation*: sharing context specific good and bad events, 2) *creative dialogue*: representants with different perspectives reflect together, and 3) *collective practice*: action-oriented approach related to the specific event [62]. By organising regular reflection meetings, or assigning roles to analyse and share lessons from reports, more meaningful and actionable learning could be enabled. Structured methods such as AI and Plan-Do-Study-Act cycles can also be parts of the LfE process [15, 16, 63]. Dialogue enables a collaborative culture and mutual understanding, which is critical for quality improvement [14, 62, 64, 65]. Learning in healthcare should not be a passive endeavour but a collaborative and often facilitated collective enterprise [14]. LfE must therefore incorporate elements that provide time and space for interprofessional reflection, fostering an environment that raises awareness of adaptive capacities [14, 66]. This should not come on top of clinical work but be planned for in work schedules [67].

Dialogue could be facilitated by daily safety briefings, weekly interprofessional meetings, or positive leadership walk-rounds. Discussions could be based on successful work (LfE-reports), or weak system components such as handoff checklists [19], and even morbidity [65]. The task is to describe tacit knowledge and work-as-done, to get in-depth knowledge and mutual understanding on how patients are kept safe [68]. Action can then be taken to help care succeed more often [68]. Hollnagel suggests that reflections on successful events should be based mostly on two adaptive capacities: 1) Anticipate: How did you recognise changes (for example, a deteriorating patient), and 2) Respond: How did you handle unexpected situations [69]. Both are relevant questions in the PACU with the potential of understanding how things usually go right, which is the crux of Safety-II [1]. However, studies have found that reflecting on and learning from what goes well is difficult, and it requires psychological safety [14, 21]. It also requires that the leader is familiar with the Safety-II underpinnings, constantly clarifies the purpose and asks in-depth questions [21].

### How do healthcare professionals experience LfE as a strategy for quality improvement?

The increased focus on organisational learning from successful work resulted in one quality improvement project during the first six months of implementation. Mandatory pre-round meetings regarding the sickest patients was a quality improvement that nurses and physicians decided to implement, based on a LfE-report. This is illustrated in the theme *Success inspires quality improvement project* and relates to the indispensable Learning and improvement dimension of PSC [3, 28]. This resembles ‘LfE-QI’, an improvement project devised in 2019 in Birmingham, in which all interventions are based on positive feedback and AI [70]. Daily reviews of gold standard antibiotic stewardship generated positive reports from the LfE team, and this significantly improved antimicrobial consumption and stewardship [12]. In ‘LfE-QI’, AI provides space for staff to reflect on what they have done well and why it went well. AI is thus a method for self-reflection, which is a prerequisite competence for a learning health system [71]. AI is also an intervention in itself because it provides positive reinforcement [12]. As Dr. Plunkett says: “If you show people what they do well, they will do more of it” [72]. Quality improvements based on work that goes well is a new approach to quality improvement, and according to WHO, this approach should be used more [3, 70].

In present study, AI could have been used more deliberately for positive reinforcement and self-reflection. Mini AI (Additional file 2) was conducted by one nurse only. The responsibility for conducting the AIs could have been shared between the entire LfE team to make it more achievable. Moreover, no one in the LfE team had experience or training in performing AI which in this intervention often took no more than 5 min. It is suggested that successful AI demands prerequisite understanding and expert facilitation [16]. However, the aforementioned LfE-QI project did not explicitly mention the presence of AI experts [12]. There, AI took 10–15 min [12]. Simply providing positive feedback, asking why processes worked and generating ideas that attempt to make a desirable event happen more frequently, have significantly improved patient outcomes [13].

In present study, as part of the reiterated process of Plan-Do-Study-Act, AI was supplemented by observing, while taking a neutral stance, and making a curious enquiry with nurses and physicians. This provided a comprehensive analysis and understanding of challenges related to pre-round meetings, and how pre-round meetings are typically conducted, i.e., work-as-done [1]. The analysis process positively influenced the quality of interaction between staff and managers and made it easier to get everyone on board and work together towards a common goal. This points to the importance of dialogue between leaders and frontline workers, which cannot be overemphasised when it comes to influencing PSC positively [73].

The healthcare professionals stated that it was more motivating to do more of something you did well. This agrees with other studies [12, 13]. This positive motivation addressed the psychology of change [38]. Celebrating and tracking improvements served as incentives and positive reinforcement, which also helped the project succeed [38].

## Strengths and limitations

This study provides an in-depth understanding of healthcare professionals’ experiences of reporting and learning from excellence, through evaluating experiences after a considerable time (six months), which contributes to increase the study’s trustworthiness [74]. *Credibility* was established by the pilot FG interviews, and by including informants with relevant and varied experiences of LfE [74]. A limitation with convenience sampling is that the volunteers may be motivated to participate because of more positive or more negative experiences with LfE than those who did not volunteer. Their narratives may therefore not reflect the general experience of LfE. However, both positive and negative experiences were shared in the discussions. The FG discussion in itself adds to the credibility of the study because what participants say can be contradicted or confirmed within the discussions [75]. The inclusion of the anaesthesiologists could be both a limitation and a strength of this study, as they had not implemented LfE in their own unit. However, they had received information about LfE and were encouraged to report successful events. It was therefore assumed that the anaesthesiologists had some experience with LfE, making their inclusion important for this study. Due to the clinical setting, the FG interviews contained only three to five informants. However, even with only three informants, FG interviews yielded rich, in-depth information. *Credibility* was also established by reading aloud a summary at the end of the FG interviews, thus ensuring that information was understood correctly [44]. *Dependability* was ensured by having the same two researchers moderating all FG interviews, and using the same interview guide. The data was collected within a relatively short period of time [44]. By using a qualitative design, we were able to uncover some of the complexities involved in the LfE intervention and gain rich insights into relevant dimensions of the PSC [28, 32]. *Confirmability* was established by including quotations that reflect the informants’ voices [44]. *Transferability* was established by presenting a detailed description of the setting, participants, the intervention, the data collection, the analytical process, and the results [44]. The findings may be relevant to similar healthcare settings.

GB’s participation in the LfE team, and her previous work in the PACU may have influenced the trustworthiness of the study [44]. She was familiar with some of the participants, although not in-depth. Participants may thus have refrained from sharing bad experiences, or they may have answered in a way they thought GB would appreciate. As this may influence the data collection process, the subject was raised before the FG interviews, and ETD moderated the LfE team discussion. GB’s prior familiarity with the PACU made it easier to arrange the data collection, because she knew the managers in the different units and had access to the room booking system. Her preunderstanding (critical care nurse, knowing how LfE was implemented, previous familiarity with the PACU culture) contributes to in-depth knowledge and understanding in the FG interviews and the analysis, serving as a potential strength to this study. This allowed GB to listen with empathy and curiosity, and to have a mutual understanding and communication with the participants. However, being part of the implementation team could cause reporting bias, where results are reported in overly positive terms. Reporting bias is a known risk when the nature of an intervention is to focus on the positive [76]. To avoid this possible bias, and to ensure reflexivity, the two moderators discussed the field notes, and all authors collaborated with their interpretation and understanding during analysis [40].

## Conclusions

Our findings offer in-depth insights into complex contextual factors when implementing LfE, shedding light on relevant dimensions of PSC. Healthcare professionals reported that LfE contributed to a positive working climate by facilitating the dissemination of positive feedback to individuals and the whole PACU. Despite this, concerns were expressed about those who did not receive any LfE-reports, raising worries about potential feelings of being unappreciated. The act of reporting successful events and fostering organisational learning proved to be challenging. However, a successful improvement project was initiated during the intervention period, inspired by a successful event.

For LfE to be worth implementing, it is essential to improve organisational learning and awareness of the system, while minimising the negative effects of LfE, such as exclusivity issues. We therefore propose that LfE must be accompanied by arenas where teams can discuss patient safety from a Safety-II perspective. We suggest that future research should include longitudinal quasi-experimental studies with comparison groups to assess the sustainability of LfE initiatives across different healthcare units, and to assess its effect on PSC dimensions such as ‘well-being’, ‘learning’, and ‘teamwork’. We also suggest that future research should explore how the LfE framework might be adapted to diverse healthcare settings, highlighting both challenges and facilitators.

## Supplementary Information

Supplementary Material 1.

Supplementary Material 2.

Supplementary Material 3.

## Data Availability

The datasets used and/or analysed during the current study are available from the corresponding author on reasonable request.

## Abbreviations

AE: Adverse events
AI: Appreciative inquiry
FG: Focus group
LfE: Learning from excellence
PACU: Postanaesthesia care unit
PSC: Patient safety culture

## Footnotes

1 ‘Closed loop’ communication is to repeat back instructions to make sure the message is understood correctly.
