# Supplementary material for: Implementing Learning from Excellence in a postanaesthesia care unit: a qualitative study of healthcare professionals’ experiences after six months
Source: BMC Health Serv Res. 2025 Apr 2;25:493. doi: 10.1186/s12913-025-12626-8 (PMC11966875; doi:10.1186/s12913-025-12626-8)
Supplement: Supplementary file 1 — Supplementary Material 1. [file 12913_2025_12626_MOESM1_ESM.docx]

**COREQ 32-item checklist for reporting of qualitative studies:**

Implementing Learning from Excellence in a postanaesthesia care unit: A qualitative study of healthcare professionals’ experiences after six months

| **Criteria for reporting:** | **Yes (Y) No (N)** |
| --- | --- |
| 1. Interviewer/facilitator Which author/s conducted the interview or focus group? | Y |
| 2. Credentials What were the researcher’s credentials? E.g. PhD, MD | Y |
| 3. Occupation What was their occupation at the time of the study? | Y |
| 4. Gender Was the researcher male or female? | Y |
| 5. Experience and training What experience or training did the researcher have? Relationship with participants | Y |
| 6. Relationship established Was a relationship established prior to study commencement? | Y |
| 7. Participant knowledge of the interviewer What did the participants know about the researcher? e.g. personal goals, reasons for doing the research | Y |
| 8. Interviewer characteristics What characteristics were reported about the interviewer/facilitator? e.g. Bias, assumptions, reasons and interests in the research topic Domain 2: study design Theoretical framework | Y |
| 9. Methodological orientation and Theory What methodological orientation was stated to underpin the study? e.g. grounded theory, discourse analysis, ethnography, phenomenology, content analysis Participant selection | Y |
| 10. Sampling How were participants selected? e.g. purposive, convenience, consecutive, snowball | Y |
| 11. Method of approach How were participants approached? e.g. face-to-face, telephone, mail, email | Y |
| 12. Sample size How many participants were in the study? | Y |
| 13. Non-participation How many people refused to participate or dropped out? Reasons? Setting | Y |
| 14. Setting of data collection Where was the data collected? e.g. home, clinic, workplace | Y |
| 15. Presence of non-participants Was anyone else present besides the participants and researchers? | Y |
| 16. Description of sample What are the important characteristics of the sample? e.g. demographic data, date Data collection | Y |
| 17. Interview guide Were questions, prompts, guides provided by the authors? Was it pilot tested? | Y |
| 18. Repeat interviews Were repeat interviews carried out? If yes, how many? | Y |
| 19. Audio/visual recording Did the research use audio or visual recording to collect the data? 20. Field notes Were field notes made during and/or after the interview or focus group? | Y |
| 21. Duration What was the duration of the interviews or focus group? | Y |
| 22. Data saturation Was data saturation discussed? | Y |
| 23. Transcripts returned Were transcripts returned to participants for comment and/or correction? Domain 3: analysis and findingsz Data analysis | N |
| 24. Number of data coders How many data coders coded the data? | Y |
| 25. Description of the coding tree Did authors provide a description of the coding tree? | N |
| 26. Derivation of themes Were themes identified in advance or derived from the data? | Y |
| 27. Software What software, if applicable, was used to manage the data? | Y |
| 28. Participant checking Did participants provide feedback on the findings? Reporting | N |
| 29. Quotations presented Were participant quotations presented to illustrate the themes / findings? Was each quotation identified? e.g. participant number | Y |
| 30. Data and findings consistent Was there consistency between the data presented and the findings? | Y |
| 31. Clarity of major themes Were major themes clearly presented in the findings? | Y |
| 32. Clarity of minor themes Is there a description of diverse cases or discussion of minor themes? | Y |

Tong, A., Sainsbury, P., & Craig, J. (2007). Consolidated criteria for reporting qualitative research (COREQ): a 32-item checklist for interviews and focus groups. *International Journal for Quality in Health Care, 19*(6), 349-357. doi:10.1093/intqhc/mzm042
