# Supplementary material for: Implementing Learning from Excellence in a postanaesthesia care unit: a qualitative study of healthcare professionals’ experiences after six months
Source: BMC Health Serv Res. 2025 Apr 2;25:493. doi: 10.1186/s12913-025-12626-8 (PMC11966875; doi:10.1186/s12913-025-12626-8)

**Additional file 2**

Mini appreciative Inquiry. Used with permission from LfE.

https://learningfromexcellence.com/resources-and-evidence/resources/


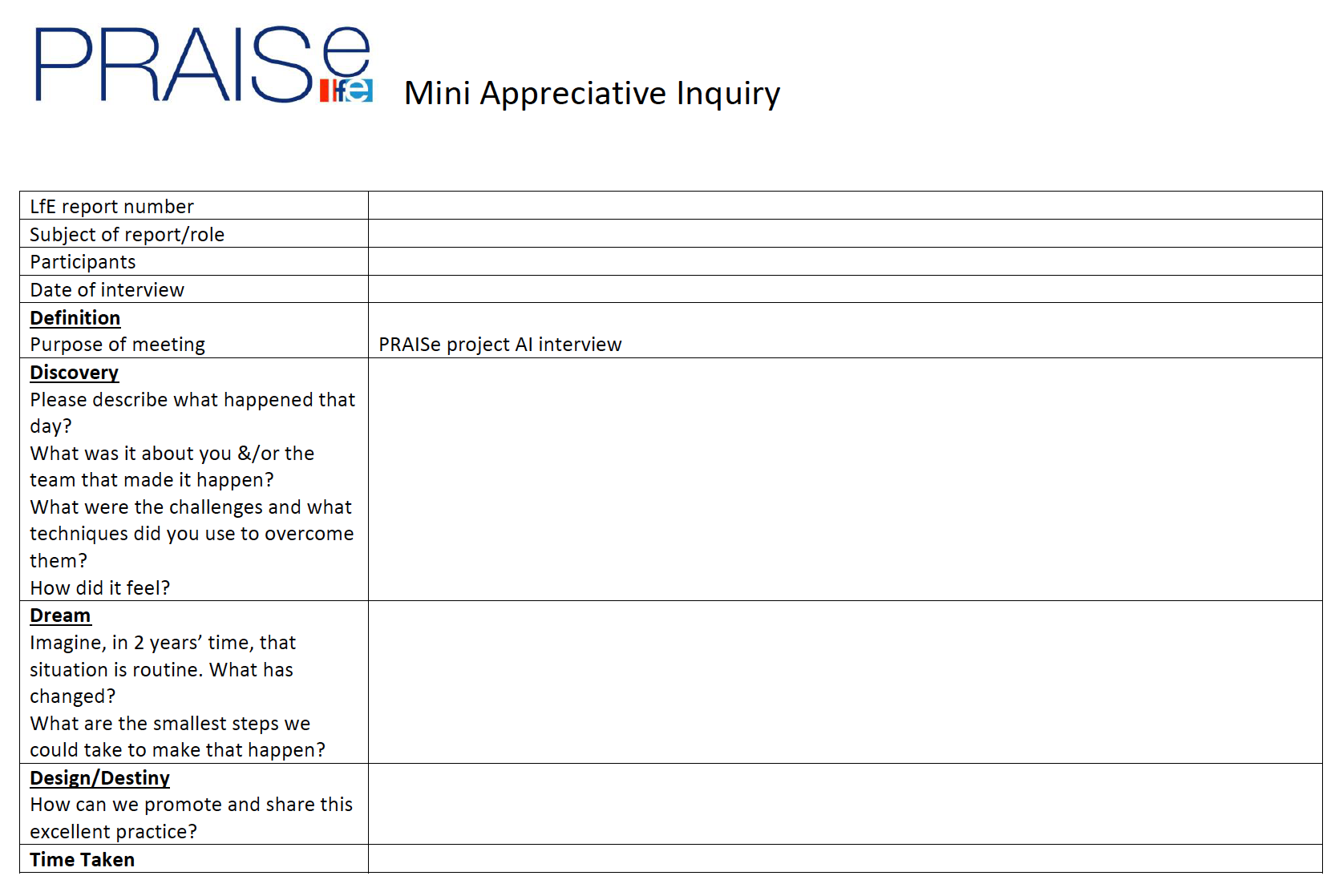

Supplement: Supplementary file 2 — Supplementary Material 2. [file 12913_2025_12626_MOESM2_ESM.docx]
