# Supplementary material for: Implementing Learning from Excellence in a postanaesthesia care unit: a qualitative study of healthcare professionals’ experiences after six months
Source: BMC Health Serv Res. 2025 Apr 2;25:493. doi: 10.1186/s12913-025-12626-8 (PMC11966875; doi:10.1186/s12913-025-12626-8)
Supplement: Supplementary file 3 — Supplementary Material 3. [file 12913_2025_12626_MOESM3_ESM.docx]

Additional file 3

**Semi-structured interview guide**

1. In what ways has the Learning from Excellence initiative been made visible in your unit?

1. What do you feel about the different ways Learning from Excellence is displayed in your unit?
2. What are your experiences with learning from successful events?
3. What are your experiences with quality improvement and changes of routines since implementing Learning from Excellence?
4. How do you perceive that implementing Learning from Excellence has affected the work culture in your unit, if at all?
5. How do you think Learning from Excellence can be further developed in your unit?

*Follow-up questions could be:*

- *Can you please elaborate?*
- *Can you please give an example?*
- *When you say.. what do you mean by that?*
- *Is it always like this?*
- *That was interesting, can you tell me more?*
- *How did that make you feel?*

In the end of the focus group interview, a brief summary is read aloud by the observer.
